# Multiple factors contribute to reproductive isolation between two co-existing *Habenaria* species (Orchidaceae)

**DOI:** 10.1371/journal.pone.0188594

**Published:** 2017-11-27

**Authors:** Wenliu Zhang, Jiangyun Gao

**Affiliations:** 1 Centre for Integrative Conservation, Xishuangbanna Tropical Botanical Garden, Chinese Academy of Sciences, Mengla, Yunnan, China; 2 University of Chinese Academy of Sciences, Beijing, China; 3 Laboratory of Ecology and Evolutionary Biology, State Key Laboratory for Conservation and Utilization of Bio-resources in Yunnan, Yunnan University, Kunming, Yunnan, China; The National Orchid Conservation Center of China; The Orchid Conservation & Research Center of Shenzhen, CHINA

## Abstract

Reproductive isolation is a key feature that forms barriers to gene flow between distinct plants. In orchids, prezygotic reproductive isolation has been considered to be strong, because their associations with highly specific pollinators. In this study, the reproductive ecology and reproductive isolation of two sympatric *Habenaria* species, *H*. *davidii* and *H*. *fordii*, was investigated by floral phenology and morphology, hand-pollination experiments and visitor observation in southwest China. The two species were dependent on insects for pollination and completely self-compatible. A number of factors have been identified to limit gene flow between the two species and achieved full reproductive isolation. Ecogeographic isolation was a weak barrier. *H*. *fordii* and *H*. *davidii* had completely overlapped flowering periods, and floral morphology plays an important role in floral isolation. The two species shared the same hawkmoth pollinator, *Cechenena lineosa*, but the pollinaria of the two orchids were attached on different body parts of pollinators. Prezygotic isolation was not complete, but the interspecific pollination treatments of each species resulted in no seed sets, indicating that unlike many other orchid species, in which the postzygotic reproductive isolation is very weak or complete absence, the post-zygotic isolation strongly acted in the stage of seed production between two species. The results illustrate the reproductive isolation between two species involves multiple plant life-history stages and a variety of reproductive barriers can contribute to overall isolation.

## Introduction

In plants, speciation is generally considered to be strongly influenced by the nature of isolating barriers [1, 2]. Reproductive isolation is a key feature that forms barriers to gene flow between distinct plant phenotypes, lineages and species, which involves a number of prezygotic and postzygotic mechanisms [3, 4]. Reproductive isolation may occur in different plant life stages and a variety of reproductive barriers contribute to overall isolation [2, 5]. Among different isolation mechanisms, floral isolation acts to prevent interspecific pollen transfer among sympatric flowering plants. It is one of the most common prezygotic isolation mechanisms and widespread in flowering plants, especially in specialized animal-pollinated plants [3, 6–9].

With more than 20000 species, Orchidaceae is one of the largest plant families in the world [10]. The great species diversity and rapid species divergence in the family are considered to be mainly driven by pollinators [11, 12]. Orchids are generally recognized to have specialized pollination system [13]. The floral isolation acts as the main reproductive isolation mechanism among sympatric species, in which orchids adapt to diverse pollinators with different body or behaviours [1, 14, 15]. Even divergent orchids share the same pollinator species, reproductive isolation also can be achieved by depositing pollinia on different body parts of same pollinator [16–19]. Pollinator specificity has traditionally been considered the main reproductive isolation mechanism in orchids [13, 20, 21]. Therefore, orchids are generally considered to have evolved strong prezygotic reproductive isolation, but very weak or no postzygotic reproductive isolation [14, 22, 23], and this can also be supported by the fact that more than 100,000 orchid hybrids have been artificially created, more than any other floricultural crop [24].

However, the plant-pollinator interactions in orchid are not always specificity. The relative importance of different types of reproductive barriers among species has become a central topic in the study of speciation [1, 2, 5, 25–26]. Although, prezygotic barriers more strongly reduce gene flow between species than postzygotic barriers [25, 27–28], in some species pairs, postzygotic isolation could sometimes be more important than prezygotic isolation, especially when species pairs share a generalized pool of pollinators [17, 29–30]. It is necessary to reassess the different stages of reproductive isolation in orchids [12].

*Habenaria* Willd. is the largest terrestrial orchid genus with approximate 880 species and is widely distributed worldwide, mainly in tropical and subtropical areas [31–33]. Flowers in the genus are often spurred on the base with available nectar, and Lepidoptera were mostly reported as pollinators [34–37], but mosquito was also found as pollinators of *Habenaria obtusata* and *H*. *parviflora* [38, 39], and *H*. *sagittifera* was pollinated by a juvenile katydid *Ducetia japonica* [40]. It is a common phenomenon that different *Habenaria* species have sympatric distributions and overlapping flowering periods [34, 41]. There are 58 *Habenaria* species found in China [42], and often, several species grow in the same place. In our field surveys on orchid species diversity in southwest Yunnan, we found that two *Habenaria* species, *H*. *davidii* and *H*. *fordii*, are sympatrically distributed with overlapping flowering periods. Therefore, we investigated the reproductive ecology of these two species. Here we present the results of our investigations, which addressed three principal questions concerning the reproductive isolation between two species: (1) What are the differences in floral morphologies and flowering phenologies between the two species? (2) What is the relative importance of different isolating barriers, especially prezygotic versus postzygotic barriers, in causing reproductive isolation between the two species? (3) Whether prezygotic isolation contributes more to total isolation than postzygotic isolation?

## Methods

### Study species and site

**Fig 1:**
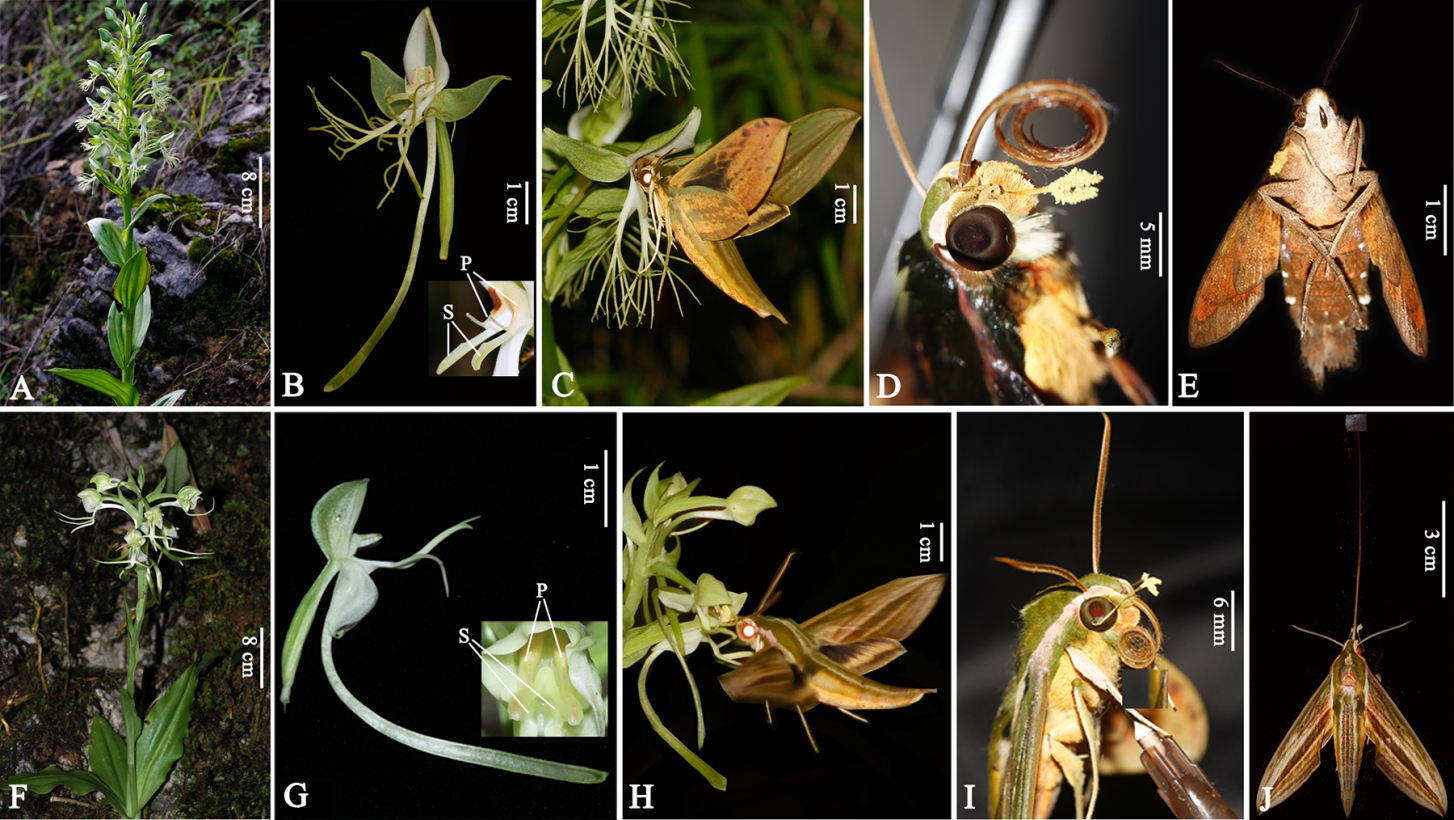
The plant, inflorescences, flowers and pollinators of *Habenaria davidii* and *H*. *fordii*. A, plant and habitat of *H*. *davidii*. B, A single flower and detail flower structure showing the position of stigma and pollinaria of *H*. *davidii*. C, *Cechenena lineosa* pollinating flowers of *H*. *davidii*. D, *C*. *lineosa* with pollinaria of *H*. *davidii* attached on its head. E, *Macroglossum fringlla*, pollinator of *H*. *davidii* with pollinaria attached on its eyes. F, plant and habitat of *H*. *fordii*. G, A single flower and detail flower structure showing the position of stigma and pollinaria of *H*. *fordii*. H, *C*. *lineosa* pollinating flowers of *H*. *fordii*. I, *C*. *lineosa* with pollinaria of *H*. *fordii* attached on its eyes. J, *C*. *lineosa*, showing long proboscis. S, stigma; P, pollinaria.

Flowers of *Habenaria* species are characterized by two stigmas with two distinct viscidia and two separate caudicles connecting each stigma to one pollinium. *H*. *davidii* and *H*. *fordii* are both small herbs with terminal racemose inflorescences (Fig 1A and 1F). Flowers of *H*. *davidii* were greenish with white lateral sepals, while the flowers of *H*. *fordii* were white with a light green dorsal petal (Fig 1B and 1G). Flowers of the two orchids were similar in structure, and the labellums of two species were all deeply three-lobed above the base, and the mid-lobe was linear, but the lateral lobes were different between species. The dorsal sepal and petals formed a hood-like structure which partially hides the column inside (Fig 1B and 1G). In both species, the two separate pollinia were concealed in their respective anther sacs. The rostellar arms of *H*. *davidii* were long and bent at the end, with both caudicles vertical upward (Fig 1B). In *H*. *fordii*, the anther was adnate to the rostellar arms, which were aclinic forward, and a suborbicular viscidium was placed at the end of each rostellar arm (Fig 1G). The stigmas were long-stalked and clavate in *H*. *davidii*, whereas those of *H*. *fordii* were short and the stigmatic surface was placed below the pollen sacs, surrounding the spur entrance (Fig 1B and 1G).

In this study, the two sympatric species were investigated in Malipo, southeast Yunnan province of China. The two species were distributed along the roadsides from Daxiechang village (23°09′N, 104°50′E; alt. 1508 m) to Shangcuandong village (23°08′N, 104°47′E; alt. 2120 m), with about 19 km of road distance but only 7.3 km direct distance. This region is a typical karst mountain landscape, and has a subtropical plateau monsoon climate with an average of 1068 mm of annual rainfall and 17.6°C of annual average temperature. Both orchids are small herbs with terminal racemose inflorescences, and grow in crevices of calcareous rocks or in thickets along the roadsides.

No specific permits were required for the described field studies, as no endangered or protected species was involved, and localities involved are not privately-owned or protected in any way.

### Ecogeographic distribution

In the study site, all flowering individuals of *H*. *davidii* and *H*. *fordii* were investigated using a GPS in 2013 to 2014. The herbarium specimens of two species were examined through Chinese Virtual Herbarium (CVH, www.cvh.org.cn) to determine the geographic distributions of the two study species.

### Floral phenology and key traits

The phenologies of *H*. *davidii* and *H*. *fordii* were monitored monthly in the study site throughout 2013 to 2014. Detailed flowering phenology of two species was observed and recorded daily by marking 25–71 individuals during the flowering seasons in 2014 and 2015. The total number of flowers per inflorescence, number of opening flowers each day, flower arrangement on inflorescences, and the flowering longevity of a single flower and inflorescence were observed or counted daily. The proportion of flowering plants per day for each species was recorded to determine the degree of the flowering period between the two species.

Flowers and inflorescences of both species were observed, photographed and described accordingly. At least 23 newly opened flowers from different individuals of each species were randomly selected to measure the flower size and separate parts using an electronic vernier caliper in 2014 (Table 1). The nectar-column heights of spurs were directly measured as an indicator of nectar volume, and the nectar sucrose concentration was measured with a hand-held, temperature-compensated refractometer (Eclipse, Bellingham & Stanley Ltd., UK) at the same time for each species.

**Table 1:** Floral traits of *Habenaria davidii* and *H*. *fordii*.

	*H*. *davidii*	*H*. *fordii*	F	P
Flower length (mm)	55.67 ± 0.92 (n = 31)	27.07 ± 0.73 (n = 23)	527.055	0.0001
Flower width (mm)	48.37 ± 0.60 (n = 31)	30.11 ± 1.02 (n = 23)	267.123	0.0001
Spur length (mm)	63.98 ± 0.91 (n = 31)	65.31 ± 1.66 (n = 23)	93.475	0.0001
Nectar volume (mm)	9.30 ± 1.09 (n = 31)	11.45 ± 1.12 (n = 23)	1.837	0.181
	(2.02–24.06)	(2.60–27.23)		
Nectar sugar concentration (%)	24.5 ± 0.44 (n = 31)	16.47 ± 0.46 (n = 23)	97.823	0.0001
	(21.5–27)	(13–19.2)		
Flowers per inflorescence	11.38 ± 0.58 (n = 72)	7.50 ± 0.49 (n = 32)	17.199	0.0001
	(4–25)	(3–15)		
Floral longevity (days)	8.71 ± 0.18 (n = 72)	12.09 ± 0.48 (n = 32)	67.165	0.0001

Data are presented as mean ± SE, and statistically homogeneous groupings based on One-way ANOVA analysis.

In order to collect floral scent of the two study species, dynamic headspace apparatus was applied to living and intact inflorescences in the study site according to the method described by Li et al. [43]. Two separated samples were collected for each species. Samples of *H*. *davidii* and *H*. *fordii* were collected from 20:00 to 21:00 on 10–11 July and on 13–14 July, 2015, respectively. All of the samples were analyzed in the Central Laboratory of Xishuangbanna Tropical Botanical Garden, Chinese Academy of Sciences. The volatiles were analyzed using an Agilent Technologies 7890A GC, equipped with an HP-5 MS capillary column (30 m × 0.25 mm; film thickness, 0.25 mm) and a mass spectrometer 5975C (Agilent Technologies, USA) as detector. Helium was used as the carrier gas, at a flow rate of 1 ml/min. Injector and detector (MS transfer line) temperatures were both 250°C. Column temperatures was gradually increased from 40°C to 100°C at 3°C/min, and increased to 200°C at 3°C/min, then programmed to 250°C at a rate of 20°C/min and held for 10 min finally. MS were recorded at 70 eV with a mass range from m/z 29 to 540. Data were analysed using the program Chemstation (G1701EA E.02.02 MSD Productivity ChemStation Software, Agilent Technologies, Germany), and the NIST spectral database within the program was implemented for preliminary identification of volatiles. The retention times and mass spectrograms of all floral volatiles were compared with those of synthetic reference compounds. Relative percentage amounts of the separated compounds were calculated automatically from peak areas of the totalion chromatogram (TIC).

### Flower visitor observation

Observations on insect visitors for each species were made during their 3 continuous flowering seasons (from 2014 to 2016). According to pre-observation in 2013, crepuscular-nocturnal visitors were observed, so observations were conducted during 17:00–23:00h for each species. To exclude the possibility of any pollinators visiting out of our observation time, randomly selected inflorescences were bagged only during observation time, and all flowers were monitored twice each day to check if removal and deposition of pollinia had occurred.

The specific observation periods and hours spent on observation for each species are detailed in Table 2. The behaviors of the flower visitors, number of flowers visited per visitation, numbers of flowers visited per inflorescence, and time length visiting on a single flower, were observed and recorded. All visitor species were photographed during observation periods. After observations were completed, we attempted to capture 5 individuals of pollinator for species identification and morphological measurements.

**Table 2:** The time of visitor observation, pollinator visiting times, the proboscis length of pollinators (mean ± SE, n = 6) and the body parts of pollinators in which pollinaria attached on in *Habenaria davidii* and *H*. *fordii*.

Species	Period	Obs. hours	Pollinators	Proboscis length (mm)	Visiting times	Pollinaria attached place
*H*. *davidii*	7–23 Jul, 2014	151 h	*Cechenena lineosa (Sphingidae)*	68.60 ± 0.84	25	head
	3–18 Jul, 2015					
	13–21 Jul, 2016		*Macroglossum fringlla (Sphingidae)*	41.25 ± 1.65	5	eyes
*H*. *fordii*	3–16 Jul, 2014	59.7 h	*Cechenena lineosa (Sphingidae)*	68.60 ± 0.84	12	eyes
	21–24 Jul, 2015					
	22–23 Jul, 2016					

Abbreviation: Obs. hours, the total observation hours.

### Hand-pollination experiments

To test self-compatibility for each species, three different hand-pollination treatments were conducted in 2014. The treatments were (i) bagging, inflorescences were bagged throughout without pollination; (ii) selfing, inflorescences were bagged before flower opening and flowers were hand-pollinated with pollinia from the same flower, and then inflorescences were bagged again; (iii) crossing, inflorescences were bagged before flower opening and flowers were hand-pollinated with pollinia of another individual, and then bagged again. The natural fruit sets of two species were investigated in 3 continuous years (from 2014 to 2016) by marking different individuals. To assess the cross-compatibility between the two species, different interspecific pollination treatments were conducted during their 3 continuous flowering seasons (from 2014 to 2016). The fruit sets of each treatment were counted at least one month later. The numbers of flowers and individuals that were used were summarized in Table 3.

**Table 3:** The fruit sets of different hand-pollination treatments.

		*H*. *davidii*		*H*. *fordii*	
Breeding system experiments		Fruit sets (%)	Inf/flo	Fruit sets (%)	Inf/flo
Hand-pollination treatments in 2014	Bagging	0	5/39	0	5/22
	Crossing	79.58 ± 11.02	12/40	89.31 ± 4.97	12/38
	Selfing	98.99 ± 1.01	11/44	98.33 ± 1.67	10/34
Natural fruit sets	2014	86.87 ± 2.38	55/545	89.76 ± 2.73	20/139
	2015	30.83 ± 6.85	13/124	85.00 ± 9.57	6/22
	2016	61.18 ± 3.65	44/478	79.40 ± 4.73	26/234
Interspecific pollination treatments	2014	17.85 ± 14.55	5/43	18.50 ± 9.60	10/40
	2015	34.78 ± 9.73	10/47	11.78 ± 4.58	15/50
	2016	16.97 ± 4.22	11/45	5.00 ± 5.00	10/31

Abbreviation: Inf/flo, Inflorescences/flowers.

The mature fruits of cross-pollination and interspecific pollination treatments were harvested two months after artificial pollination during September (from 2014 to 2016), and the seed viability of interspecific pollination treatments in the two species was determined by observing the presence of viable embryos under an optical microscope and comparing with the seeds of cross-pollination treatments for each species.

To test pollen germinability in vitro, twenty-five flowers were subjected to interspecific pollination and twenty-five to cross-pollination for each species. After 4, 8, 12, 24 and 48h or 72h, each stigma was sectioned and softened in 0.1M NaOH at 60°C for 1h. Afterward, the stigmas were incubated with 0.1% aniline blue in phosphate buffer (Ph 8.3) for 48h. A total of 40 slide preparations for each species were examined under a fluorescence mircroscope with blue excitation (410 nm) to observe pollen tube formation.

### Statistical data analyses

The floral size (length and width), the length of spur, the floral longevities, the number of flowers in single inflorescence (mean flower number per inflorescence) and the fruit success (mean fruit set per inflorescence) of the two study species were statistically compared using an One-way ANOVA test (Unequal sample size, equal variance). All statistical analyses were performed by SPSS ver. 22.0 for Windows (SPSS Inc., Chicago, IL, USA).

## Results

### Ecogeographic distribution

In our study site, 90 flowering plants *of H*. *davidii* between altitudes of 1760 to 2120 m and 57 flowering plants of *H*. *fordii* between altitudes of 1508 to 1800 m were found along the roadsides in 2014. That is to say that the *H*. *fordii* distribution overlapped with *H*. *davidii* from 1760 to 1800 m in altitude. The results of herbarium specimen examination showed similar patterns, with *H*. *davidii* recorded from 1339 to 3200 m (Mean = 2170 m, *n* = 53) and *H*. *fordii* from 106 to 2200 m (Mean = 1106 m, *n* = 31).

### Floral phenology and key traits

**Fig 2:**
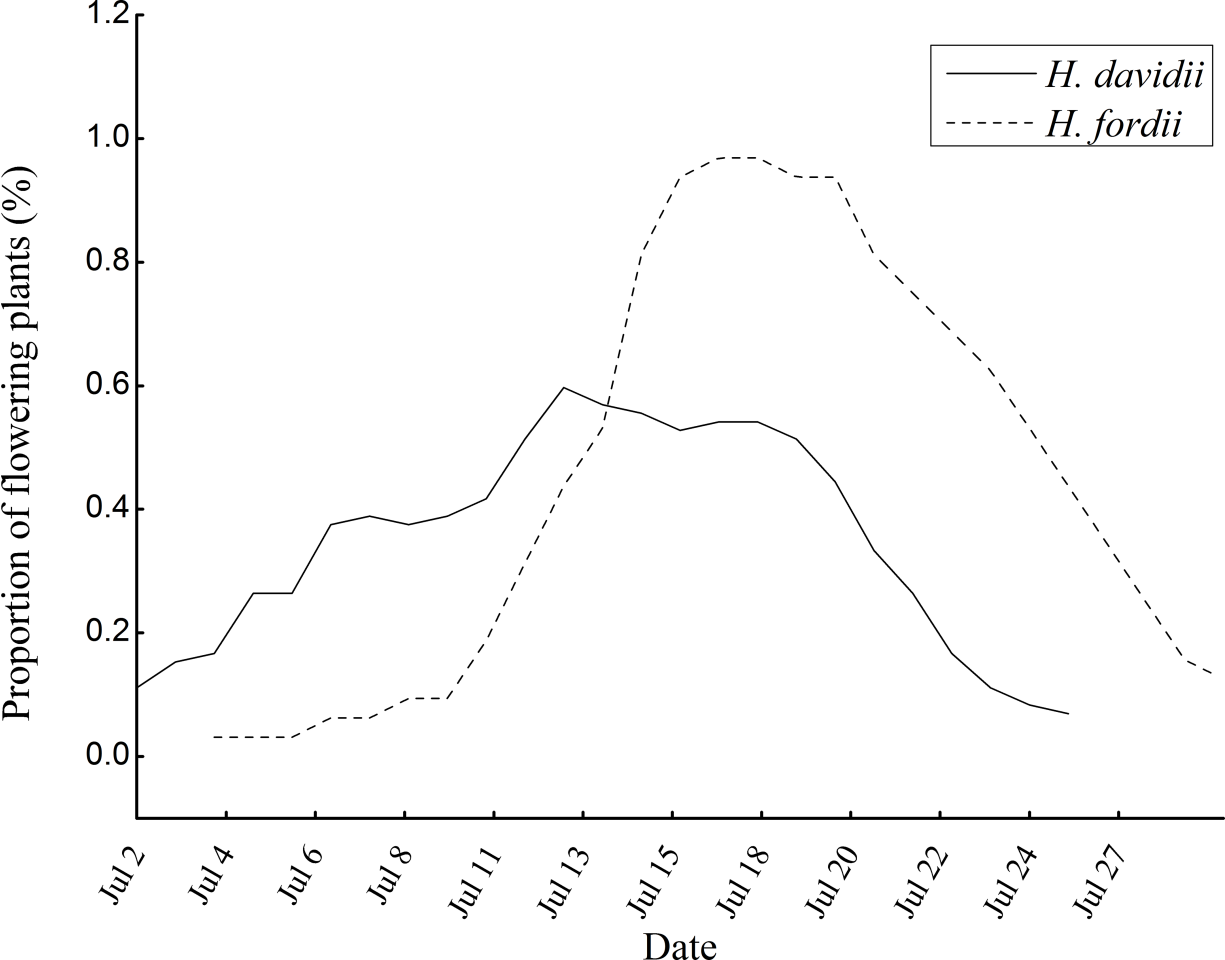
Flowering phenology (The proportion of flowering plants per days) of *Habenaria davidii* and *H*. *fordii*. One census per day from 2 to 30 July, 2014.

The two orchids flowered in July in our study site. In 2014, the flowering periods of *H*. *davidii* (2–26 July) and *H*. *fordii* (4–30 July) almost completely overlapped (Fig 2). For both species, at least five fleshy leaves extend flat on the ground with racemose inflorescences at the top of plants. The inflorescences of *H*. *davidii* were 54.38 ± 2.24 (*n* = 17) cm tall with 4–25 flowers, while those of *H*. *fordii* were 32.23 ± 2.02 (*n* = 15) cm tall with 3–15 flowers. Flowers of two species were tidily or loosely arranged on the inflorescences, and opened gradually from the bottom to the top (Table 1, Fig 1A and 1F). The floral longevities of both species were around one week, but the inflorescences of *H*. *fordii* last significantly longer than *H*. *davidii* (*F* = 67.165, *df* = 1, *P* < 0.001; Table 1).

Overall, flowers of both species varied significantly on size, and the flower of *H*. *davidii* was significantly larger than *H*. *fordii* (length: *F* = 527.055, *P* < 0.001; width: *F* = 267.123, *P* < 0.001; Table 1). Flowers of the two species had a pendulous and cylindric spur on the base with obvious nectar inside. The nectar volume and sugar concentration for each species were subject to remarkable variation (Table 1).

From 18:30 hours, a sweet fragrance which can be detected obviously by a human nose was produced from the two orchid flowers, and by 20:00 hours it could be detected from several meters away. To omit the compounds detected only in trace amounts (less than 0.1), we found a total of 11 main compounds in the headspace of both species. There was a complete overlap in floral compounds produced, as all of the volatiles in *H*. *fordii* were found in *H*. *davidii*. However, the relative amounts of active compounds differed between two species (S1 Table). The scent of *H*. *davidii* was composed chiefly of methyl benzoate, linalool, and dodecane, while linalool and dodecane were the main compounds in *H*. *fordii* (Table 4).

**Table 4:** The relative content of volatile compounds of *H*. *davidii* and *H*. *fordii*.

*H*. *davidii*		*H*. *fordii*	
Compounds	Relative content (%)	Compounds	Relative content (%)
Methyl benzoate	49.29	Linalool	75.894
Dodecane	15.06	Dodecane	10.873
Linalool	3.05	Decane	1.256
Methyl salicylate	2.76	*(E*,*E)*-2,6-Dimethyl-1,3,5,7-octatetraene	0.713
*(E)-*Ocimenol	1.76	α-Isophporone	0.214
Benzyl alcohol	1.64	Total	88.95
Decane	0.76		
*(E*,*E)-*2,6-Dimethyl-1,3,5,7*-*octatetraene	0.58		
Geraniol	0.42		
Benzaldehyde	0.38		
α-Isophporone	0.28		
Total	75.97		

### Pollinator observation

Totally, 151h of visitor observations were achieved on *H*. *davidii*, and three insects were observed visiting flowers during our observations. *C*. *lineosa* (Sphingidae) visited flowers of *H*. *davidii* regularly and was considered as the main pollinator (Fig 1C). It hovered in the front of the flowers and inserted its proboscis into the spur. Its head pressed against floral column and then pollinaria were attached on its head (Fig 1C and 1D). *C*. *lineosa* spent 4–6 s (*n* = 11) on one flower and visited 2–3 flowers (*n* = 6) per visitation. Its proboscis length was 68.60 ± 0.84 mm (*n* = 6), which was shorter than the spur length of *H*. *davidii* (80.68 ± 0.62 mm, *n* = 31). *Macroglossum fringlla* (Sphingidae) was occasionally observed visiting flowers of *H*. *davidii* and can took away the pollinaria attached on its eyes (Fig 1E). Its proboscis length was 41.25 ± 1.65 mm (*n* = 6), which is much shorter than spur length of *H*. *davidii*. Another visitor we observed was *Agrius convolvuli* (Sphingidae). It visited flowers of *H*. *davidii* frequently and usually appeared during 21:00 to 22:00h which was later than *C*. *lineosa* and *M*. *fringll*. The visiting behavior of *A*. *convolvuli* was similar to *C*. *lineosa*, but it can’t touch with flower column because its proboscis was considerably longer (109.51 ± 2.25 mm, *n* = 6) than flower spur. We didn’t observe it removing pollinaria and the flowers remained intact after visits. All three visitors were very sensible to the weather, and never appeared in the windy and cloudy night. We record 23 times visits of *C*. *lineosa* and 31 visits of *A*. *convolvuli* during totally 42 days of observation from 2014 to 2016.

The only visitor to flowers of *H*. *fordii* we observed was *C*. *lineosa*, and it was also the efficient pollinator (Fig 1H). Its visiting behavior to *H*. *fordii* was same as when visiting to *H*. *davidii*, and the pollinaria were attached on the eyes (Fig 1I). The proboscis length of *C*. *lineosa* was well match with the spur length of *H*. *fordii* (65.13 ± 1.65mm, *n* = 23; Fig 1J). We record 12 visits of *C*. *lineosa* in 20 days of observation during 2014 to 2016.

For all inflorescences bagged only during observation time, no pollinia removal or deposition had occurred, indicating that no insect visitation to both species out of our observation time.

### Hand-pollination experiments

In our hand-pollination treatments, no fruit was found in the bagging treatments, suggesting that spontaneous autogamy did not occur in either orchid species. The fruit sets of the selfing, crossing and natural pollination treatments were not significantly different for the two species in 2014 (*H*. *davidii*: *F* = 2.642, *df* = 2, *P* = 0.119; *H*. *fordii*: *F* = 2.518, *df* = 2, *P* = 0.128; Table 3). The natural fruit sets kept stable from 2014 to 2015 for *H*. *fordii* (*F* = 1.486, *df* = 2, *P* = 0.236; Table 3), but were significantly different among years for *H*. *davidii* (*F* = 43.061, *df* = 2; *P* < 0.001; Table 3), in which the natural fruit set was higher in 2014 than in 2015 and in 2016.

For the interspecific pollination treatments, the fruit sets were significantly lower than in crossing treatments both for *H*. *davidii* (*F* = 72.875, *df* = 1, *P* < 0.001) and *H*. *fordii* (*F* = 67.067, *df* = 1; *P* < 0.001) in 2014, respectively. However, the fruit sets of interspecific pollination treatments kept stable between studied years (from 2014 to 2016) for the two species (*H*. *davidii*: *F* = 1.480, *df* = 2; *P* = 0.249; *H*. *fordii*: *F* = 0.975, *df* = 2; *P* = 0.388) (Table 3).

We observed seeds from cross pollination using an optical microscope and determined that 67.48% ± 3.90% (*n* = 10 fruits; *H*. *davidii*) and 60.85% ± 5.47% (*n* = 10 fruits; *H*. *fordii*) of them contained viable embryos. We didn’t harvest any seeds for interspecific pollination treatments of each species because all fruits aborted before they matured.

**Fig 3:**
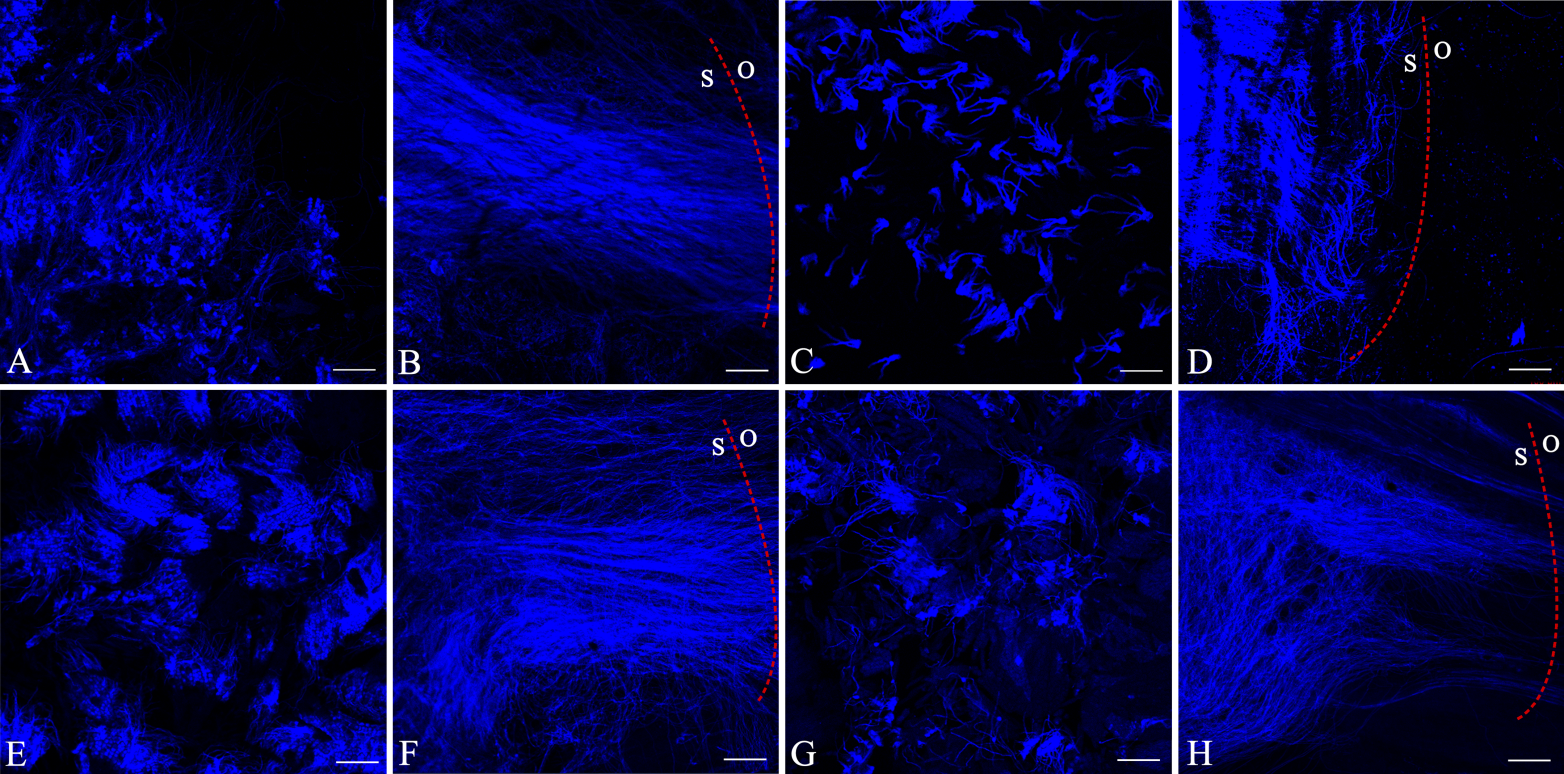
Fluorescence microscopy image of pollen tube growth in *Habenaria davidii* (A-D) and in *H*. *fordii* (E-H). A-B, pollinaria germination after 4h and 48h of crossing pollination in *H*. *davidii*. C, pollinaria germination after 12h of interspecific pollination in *H*. *davidii*. D, pollinaria germination after 48h of interspecific pollination in *H*. *davidii*, showing pollen tubes were arrested in the stigma. E-F, pollinaria germination after 12h and 48h of crossing pollination in *H*. *fordii*. G, pollinaria germination after 12h of interspecific pollination in *H*. *fordii*. H, pollinaria germination after 48h of interspecific pollination in *H*. *fordii*, showing a small number of pollen tubes reached the ovary. The length of the scale is 200um; S, stigma; O, ovary.

In the pollen tube growth experiments, we observed pollen tubes emerging from the massulae of all the samples after only 8h of incubation, whether from cross pollination or interspecific pollination experiments from both species. Nonetheless, different amount and growth rate of pollen between cross pollination and interspecific pollination experiments were evident after 12h and 48h of incubation (Fig 3). Indeed, microscopic inspections of *H*. *davidii* in cross pollination revealed that pollen tubes grew in the stigma and reached the ovary within 48h (Fig 3A and 3B), but most of pollen tubes in interspecies became arrested in the lower portion of the stigma only a small number of pollen tubes from interspecific pollination reached the ovaries within 72h (Fig 3C and 3D), and two out of five interspecific samples failed to reach the ovaries even in 72h. However, all the samples from *H*. *fordii* showed that a larger number of pollen from cross pollination and a small number of pollen from interspecific pollination reached the ovaries within 48h (Fig 3E–3H).

## Discussion

Plant speciation is a continuous process [44], and multiple isolating mechanisms act sequentially at different stages. Each isolating barrier can prevent only the potential gene flow that was not already eliminated by earlier acting barriers, and therefore, early barriers would contribute more to reproductive isolation than late barriers [2, 45, 46]. Geographic isolation was considered as the first opportunity to limit gene flow in the sequence of barriers that can act to separate species [47, 48]. However, ecogeographic isolation was not a strong barrier for our studied two *Habenaria* species. As suggested by the results of herbarium specimen examination and our field investigation, the two study species were sympatrically distributed but varied in altitudinal ranges. *H*. *fordii* distributed overlap with *H*. *davidii* in altitude from 1760 to 1800m.

*H*. *davidii* and *H*. *fordii* also had completely overlapping flowering periods in July, so there is no temporal barrier to gene exchange (Fig 2). It is common in Orchidaceae that several co-existing species flower at same time, and floral isolation has been demonstrated to be a crucial isolating barrier among those species [1, 15, 49, 50]. Floral isolation can be achieved by subtle differences in floral morphology, which worked by positing the pollen on different body parts of pollinator [28, 51, 52] or attracting different kinds of pollinators [53, 54]. In this case, although the gynostemium of both *Habenaria* orchid species are very similar in shape and size, and easily approached by visiting insects, the differences in stigma position and pollinarium shape between *H*. *davidii* and *H*. *fordii* suggest that different pollination mechanisms may be involved.

*H*. *davidii* and *H*. *fordii* show a clear suite of floral adaptation to hawkmoth pollination, including a long-spur with nectar production, white or light green color, and fragrance emission in the dusk. Hawkmoths are apparently attracted to flowers from a distance by scent, and as they approached the flowers, they are guided by both odour and visual cues [55]. Different pollinators may have different innate preferences for certain odour compounds [56, 57], but pollinators may also learn floral odour bouquets and use this to maintain flower constancy [58]. In our study, linalool was the main floral scent compounds of *H*. *fordii*, while the floral scent of *H*. *davidii* was a complex blend of compounds. There was a complete overlap in floral compounds produced, as all of volatiles in *H*. *fordii* were found in *H*. *davidii*. However, the floral odour may fluctuate temporally, and more study should be done to compare the compounds and quantity between the two species. Here, we suggest that the linalool may be the essential compound to attract pollinator *C*. *lineosa*. However, given that often not all floral odour compounds have a signal function with respect to pollinators [59], and there are more compounds in the floral scent of *H*. *davidii*, it is possible to attract more visitors for those flowers.

Floral isolation has been suggested to be strong in orchid, because their associations with pollinators are often highly specific [13]. In some orchids with overlapping flower periods, interspecific pollen transfer is avoided by individual pollinators visiting only one orchid species [53, 58]. This flower constancy is considered to be a very common type of isolation mechanism [14, 17]. In this study, *H*. *fordii* and *H*. *davidii* shared the same pollinator, *Cechenena lineosa*, but the pollinaria were attached on the different body parts. This shows that floral isolation via mechanical isolation was a means of preventing pollen transfer between these two species. The differences in the size and morphology of the flower stigma and pollinaria have an important effect on pollen transfer. The proboscis of *C*. *lineosa* could only partly enter the spur of *H*. *fordii* flowers but could completely enter the longer spur of *H*.*davidii* flowers. Flower size, spur, and column morphology are most important for morphological isolation, but flower constancy seems not to be an effective isolating barrier between the two species, since the same individual hawkmoth was sometimes seen visiting both species. Therefore, considering the flower spurs were varied with length between individuals, floral isolation does not work all the time.

In the hand-pollination treatments, the lack of fruit production in the all bagging treatments indicated that both *Habenaria* species were dependent on insects for pollination. The fruit sets of the selfing, crossing and natural pollination treatments were not significantly different for both species, suggesting that both species were completely self-compatible and fruit production under natural conditions were not suffered pollinator-limitation.

The pollen tube growth experiments have shown that growth of interspecific pollen tubes is restricted to stigmatic cell layers in some *H*. *davidii*, and the amount of pollen tubes which reached to ovary was decreased in *H*. *fordii*. The findings of this study clearly demonstrate that a strong post-pollination, prezygotic isolation mechanism exists at the level of the pollen-stigma interaction in *H*. *davidii*, and postzygotic isolation acts as embryo and seed mortality in *H*. *fordii*. In the interspecific pollination treatments, we observed a few fruits two months after hand-pollination (Table 3), but all fruits aborted before maturity and no seeds were found in cross-pollination treatment between *H*. *davidii* and *H*. *fordii*. A high abortion rate of fruits and the formation of seeds lacking embryos have been documented in interspecific manual crosses, suggesting that strong post-zygotic isolation occurred between *H*. *davidii* and *H*. *fordii*.

Most plant species are separated by a number of isolation mechanisms that form barriers to gene flow [3]. The reproductive isolation involves multiple plant life-history stages and a variety of reproductive barriers contribute to overall isolation [2, 5, 25]. In this study, a number of factors have been identified that limit gene flow between the two closely co-existing *Habenaria* species and achieved full reproductive isolation. This explains why we did not find any potential hybrids in nature. In contrast to most orchids, prezygotic isolating mechanisms have been found to be strong, and postzygotic barriers contribute less to total isolation when fruit and seed with embryos from interspecific crossing experiments are available [15, 60]. Our resulted confirmed that both prezygotic and postzygotic played important roles in maintain the integrity of the two co-existing species.

## Supporting information

S1 Table TIC profile of floral odour for *Habenaria davidii* and *H*. *fordii*. (XLSX)
